# Immunopathology in schistosomiasis is regulated by TLR2,4- and IFN-γ-activated MSC through modulating Th1/Th2 responses

**DOI:** 10.1186/s13287-020-01735-2

**Published:** 2020-06-05

**Authors:** Chao Liu, Yi-shu Zhang, Fang Chen, Xiao-ying Wu, Bei-bei Zhang, Zhong-dao Wu, Jun-xia Lei

**Affiliations:** 1 Department of Parasitology of Zhongshan School of Medicine, Sun Yat-sen University, Guangzhou, China; 2 Department of Parasitology of Guilin Medical University, Guilin, China; 3 School of Medicine, South China University of Technology, Guangzhou, China; 4 The Third Affiliated Hospital, Sun Yat-sen University, Guangzhou, China

**Keywords:** Liver fibrosis, Mesenchymal stem cell, Schistosomiasis, Hepatic granulomas, TLR, Immune modulation

## Abstract

**Background and aims:**

A marked egg-induced CD4^+^ T cell programmed inflammation and subsequent hepatic fibrosis characterize the pathogenesis of schistosomiasis. Mesenchymal stem cell (MSC) has been extensively studied for the treatment of schistosomiasis. However, the mechanism by which MSCs modulate the pathogenesis of schistosomiasis has not been clarified. Furthermore, the local inflammatory milieu may greatly influence the immunoregulatory properties of MSCs, and our early experiments demonstrated that Toll-like receptor (TLR)2/TLR4 agonist effected immune modulation of MSC. Here, we further investigated their modulation on the pathogenesis of schistosomiasis.

**Methods:**

Adult BALB/c male mice were percutaneously infected with 16 ± 2 pairs *S. japonicum* cercariae and received intravenously pretreated MSC at 1 week and 3 weeks post-infection, respectively. At 8 weeks post-infection, effects of MSC on liver histology were shown by hematoxylin and eosin (H&E) staining and Masson staining and quantitatively compared by the hepatic hydroxyproline content; α-smooth muscle actin (α-SMA), collagen type I(Col-1), transforming growth factor β (TGF-β), and tumor necrosis factor-α (TNF-α) gene expression in the liver were assessed by semi-quantitative polymerase chain reaction (PCR); the Th1/Th2 dominance among different groups was compared by analyzing CD4^+^ interferon-γ (IFN-γ)+ and CD4+interleukin-4 (IL-4)+T cells in the liver by flow cytometry and serum level of IFN-γ and IL-5 using enzyme-linked immunosorbent assay (ELISA). Effects of different kinds of MSC were further evaluated in vitro by the coculture system.

**Results:**

Results showed TLR4- and IFN-γ-activated MSC alleviated liver fibrosis in infected mice, without a significant increase of mortality, and unpretreated MSC showed no clear improvement; however, TLR2- and IFN-γ-activated MSC displayed aggravated immunopathology. In accord with the pathological results, TLR4- and IFN-γ-activated MSC groups showed moderate enhancement of Th1 response in vitro and clear Th1 dominance in vivo without leading to extreme inflammation, whereas TLR2- and IFN-γ-activated MSC not only induced Th1 response, but also triggered excessive inflammation as evidenced by atrophy of the thymus and higher TNF level in the coculture system.

**Conclusions:**

This study demonstrates that TLR4 combined with IFN-γ can activate the MSC group with positive effects on the pathology of schistosomiasis by modulating Th subsets at some degree. This result suggests that when MSC is being used to treat different immuno-disturbance complications, subtle pretreatment methods should be seriously considered.

## Introduction

Schistosomiasis is one of the world’s major public health problem in terms of morbidity and mortality, which is characterized by a marked egg-induced CD4^+^ T cell programmed inflammation and subsequent fibrosis [1, 2]. During infection, an initial proinflammatory Th1-type polarized response is continuously triggered by *Schistosoma*-soluble adult worm antigen fractions, with elevated IFN-γ, TNF-α, and IL-12 levels, and then it is rapidly driven by egg antigens to a Th2-type dominant response (at approximately 6 weeks post-exposure). This is believed to mediate granuloma progression and following fibrosis [3]. On the other hand, this response also benefits the host by alleviating inflammation damage to the liver and intestine. However, it also leads to retardation of pathogen eradication and underpinned refractory chronic conditions [4]. How to establish finely regulated T subsets responses at different stages is the key issue in the treatment of schistosomiasis.

Recently, based on widely anti-inflammatory effects, MSC has been employed to attenuate hepatic fibrosis induced by S*chistosoma* infection, and in parallel with the decreased hepatic stellate cell (HSC) activation and enhanced liver regeneration [5–7]. However, the effects of MSC on T cells, especially on Th1/Th2 modulation in this model, are still largely unknown. MSCs broadly suppress T cell activation and proliferation in vitro via a plethora of soluble and cell contact-dependent mediators, such as TGF-β, prostaglandin E2 (PGE2), IDO (human), nitric oxide (NO, mice), hepatocyte growth factor (HGF), and jaggd-1 [8, 9]. In terms of modulation on Th subsets, it was reported that MSC inhibits disease-associated Th1, Th2, and Th17 cells or restore new Th1/Th2 balance [8, 10, 11]. Furthermore, under the local inflammatory milieu, cytokine or pathogen-associated molecular patterns (PAMPs) (such as TLR ligands) may greatly influence the immunoregulatory properties of MSCs and thereby impact the outcome of MSC-based therapies [12, 13]. It was reported that TLR3/TLR4 ligated MSC either suppressed or enhanced Th1/Th17 response, respectively, thus had different roles in an experimental autoimmune encephalomyelitis (EAE) disease model [14]. Moreover, some reports even suggested that in vitro conditioning of MSCs by suitable TLR ligands could boost the effectiveness of MSC and thereby lead to more effective and greater homogenous therapies in a clinical context [15–17]. So, we questioned whether TLR2/TLR4-ligated MSC could also modulate Th1/Th2 responses and thus have different roles in the schistosomiasis model. Therefore, we investigated the effectiveness of both MSC and the TLR2/4-ligated MSC to regulate Th1/Th2 response in the schistosomiasis model, which will provide new information about the potential of MSC-based therapies in the treatment of *Schistosoma* egg-induced liver pathology.

## Materials and methods

### Host animals

C57BL/6 or BALB/c male mice, 6–8 weeks old, were obtained from the Laboratory Animal Center of Guangdong Province (Guangzhou, China) and kept in specific pathogen-free environments in the Animal Care Facility of Sun Yat-sen University.

### Isolation and culture of mouse MSCs

Mouse MSCs were isolated from the bone marrow of 8-week-old C57BL/6 mice according to the improved low-density culture method reported previously [18]. In brief, the femurs and tibiae were removed and placed on ice in 5 ml l-Dulbecco’s Modified Essential Medium (DMEM) complete medium. Each bone marrow cavity was flushed with the medium, and individual cells were obtained by filtration through a 70-μm cell strainer. After red blood cells were removed by ammonium chloride lysis, the remaining cells were washed with Hanks Balanced Salt Solution (HBSS), resuspended in l-DMEM complete medium, and plated in culture flasks at the low density of 5 × 10^4^ cells/cm^2^. The cells were then cultured for 3 days, and non-adherent cells were removed by a complete change of the medium, while the remaining adherent cells were cultured continuously. After 3 passages, MSCs were flow cytometrically characterized by being positive for Sca-1, CD106, and CD44, but negative for CD45 and CD11b.

### Pretreatment of MSCs with TLR2/4 ligand combined with IFN-γ

MSCs with 70% confluency were incubated with mouse IFN-γ (20 ng/ml, R&D Systems, Minneapolis) plus Pam_3_Cys (TLR2 ligand, 20 ng/ml) (Merck, Calbiochem) or LPS (TLR4 ligand, 10 ng/ml) (Sigma-Aldrich, St Louis) for 10 h, and then the MSCs were collected and thoroughly washed two times for further in vitro or in vivo experiment.

### Experimental infection and MSC administration

BALB/c male mice were divided randomly into 5 groups: normal group (uninfected), infected control group, MSC group, TLR2 plus IFN-γ-treated MSC group, and TLR4 plus IFN-γ-treated MSC group. Each group included ten mice. Mice were percutaneously infected with 16 ± 2 pairs cercaria collected from *S. japonicum*-infected snails, which were bought from the Jiangsu Institute of Parasitic Diseases (Wuxi, China). At 1 week and 3 weeks post-infection, mice received via the tail vein 5 × 10^5^ MSC or pretreated MSC suspended in 0.2 ml of saline. Clinical evaluation of the disease was monitored based on body weight and mortality rate (the data from four repeated experiments were analyzed).

### Histological evaluation

The thymus, spleen, and the remaining right liver lobe of each mouse were fixed in 4% paraformaldehyde, embedded in paraffin, and cut into about 5-μm sections. The sections were stained with hematoxylin and eosin (H&E). Representative H&E-stained thymus, spleen, and liver sections from each animal were scanned under × 200 magnifications with a compound microscope. The liver granuloma was quantified by calculating the sum of the granulomatous area using AxioVision Rel 4.7 (Carl Zeiss GmbH, Jena, Germany).

### Evaluation of fibrosis

The hepatic fibrosis was observed by Masson stainings to show the deposition of collagen fibers in the liver at 8 weeks post-infection and quantified by measurement of hydroxyproline levels and Acta2, Tgfb, and Col1a1 mRNA expression by real-time PCR.

### Real-time polymerase chain reaction

Total RNA was extracted from liver tissue using TRIzol reagent (Invitrogen, Life Technologies Carlsbad, CA, USA). The cDNA was synthesized with the PrimeScript RT reagent kit (Takara, Otsu, Shiga, Japan) according to the manufacturer’s protocol. PCR was performed on the LightCycler480 (Roche, USA) using primers specific for Acta2, Col1a1, Tgfb, and Tnf listed in Table 1. Amplification was performed in a total volume of 20 μl for 40 cycles, and products were detected using SYBR Green I dye (Roche Life Science, Switzerland). The housekeeping gene Gapdh was used as an internal control, and quantitation of relative mRNA expression was calculated using the 2^−ΔΔCt^ method.

**Table 1:** Primer sequences of Gapdh, Acta2, Tgfb, Tnf, and Col1a1 genes used in the RT-PCR

		Sequence (5′→3′)
Gapdh	Sense	GGCAAATTCAACGGCACAGT
	Anti-sense	AGATGGTGATGGGCTTCCC
Col1a1	Sense	CCTGGCAAAGACGGACTCAAC
	Anti-sense	GCTGAAGTCATAACCGCCACTG
Acta2	Sense	TGACCCAGATTATGTTTGA
	Anti-sense	GCTGTTATAGGTGGTTTCG
Tgfb	Sense	TGACGTCACTGGAGTTGTACGG
	Anti-sense	GGTTCATGTCATGGATGGTGC
Tnf	Sense	GGTGCCTATGTCTCAGCCTCTT
	Anti-sense	GCCATAGAACTGATGAGAGGGAG

### Flow cytometry detection

#### Intracellular cytokine staining

Percentages of Th1 (CD3^+^CD4^+^IFN-γ^+^) and Th2 (CD3^+^CD4^+^IL-4^+^) cells in the liver of Babl/c mice at 8 weeks post-infection were detected by flow cytometry. Liver mononuclear cells were isolated from the livers of mice as previously described [19]. Briefly, mouse livers were minced through a 100-μm cell strainer (BD Falcon) and re-suspended in PBS (pH 7.2) and centrifuged at 50×*g* for 5 min. Supernatants containing liver mononuclear cells were collected and washed in PBS. The cell pellets were re-suspended in 35% Percoll solution (Sigma-Aldrich) and gently overlaid onto 70% Percoll. After being centrifuged (600×*g*) for 30 min at room temperature, the inter-phase containing liver mononuclear cells were collected and washed twice in PBS. These cells were subsequently used for stimulation and surface staining.

Then, lymphocytes were stimulated with phorbol 12-myristate 13-acetate (PMA) (50 ng/ml) and ionomycin (500 ng/ml) for 6 h; during this period, brefeldin A (BFA; 10 μg/ml) was used to inhibit the secretion cytokines (all from Sigma Aldrich). Collected cells were stained with anti-mouse CD3e-PE-Cyanine7, CD4-BV605, and CD8-Pacific Blue. Subsequently, the cells were washed, fixed, and permeabilized with Cytofix/Cytoperm buffer and stained with anti-mouse IFN-γ-PerCP-Cyanine5.5 or IL-4-PE (or isotype IgG2a control antibody) (eBioscience, San Diego, CA, USA) following the manufacturer’s instruction. Stained cells were detected by flow cytometry (Becton Dickinson), and the data were analyzed using the FlowJo7.6 software.

### ELISA

For cytokine level assay in culture supernatants, spleen lymphocytes from mice at 8 weeks post-infection were prepared. Briefly, single-cell suspensions were prepared by collecting the cells after the spleen was ground in incomplete Roswell Park Memorial Institute (RPMI) 1640 medium (Gibco, USA), followed by red blood cell lysis, washing by staining buffer which contained phosphate-buffered saline (PBS)with 1% fetal bovine serum (FBS), and then filtering through a 200-gauge mesh. Splenocytes (5 × 10^6^ cells/well) were cultured with or without 10 μg/ml soluble egg antigen (SEA) in the absence or in the presence of unpretreated MSC, TLR2+IFN-γ-treated MSC, or TLR4+IFN-γ-treated MSC for 72 h at 37 °C in 5% CO_2_. The concentrations of IFN-γ, IL-5, and TNF in cell culture supernatants were determined by ELISA.

The level of cytokine in the serum or culture supernatants was determined according to the instruction of the ELISA Kits (eBioscience). In brief, mouse IFN-γ, TNF, IL-5, and IL-10 were detected by biotinylated monoclonal antibodies, which were evidenced by avidin-conjugated horseradish peroxidase followed by incubation with TMB substrate. OD values at 450 nm were recorded using the MK3 microplate reader.

### Assessment of worm and egg burdens

After 8 to 9 weeks of infection, parasite burden including eggs and adult worms was identified. The worms were collected and counted through perfusion of the portal vein with PBS. In addition, the intestinal tract of each mouse was also examined for residual worms. Eggs in the liver were calculated after putting 0.2 mg of liver in 10% KOH overnight and counting the number of eggs by taking 10 μl three times.

### Statistical analysis

All analyses were carried out with the SPSS 21.0 software. Data were shown as mean ± SD. Multiple comparisons were performed by one-way ANOVA and followed by LSD post-test for comparison between the two groups. Significance was considered when *P* < 0.05. We used GraphPad Prism 5.01 software (GraphPad Software, USA) for all graphical representations.

## Results

### TLR2 ligand or TLR4 ligand combined with IFN-gamma effected some key mediator expression of MSC after a short-time coculture

Mouse MSCs were isolated from bone marrow aspirates by adherent culture. The characteristics of the MSCs were confirmed based on the expression of related surface markers. They expressed a panel of surface markers, including Sca-1, CD44, and CD106, and the absence of CD45 and CD11b (Figure s1). Since many reports have shown that MSC activated by inflammatory cytokine could function more obviously and more homogenously, and a series of modulators (such as iNOS, PGE_2_, TGF-β, IL-6, IFN-β) could contribute to the immunomodulatory property of MSC, we investigated the effects of TLR2/4 and/or IFN-gamma activation on these factor expression of MSC. Our results showed that TLR2 ligand or TLR4 ligand combined with IFN-gamma clearly increased Nos2, Il6, and Ifnb expression of MSC after a short-time coculture, while no significant increase on the expression of Jag1, Ptgs2, and Tgfb (Figure s2). Their immunoregulatory capability in vivo was further investigated in the infection model with distinct Th1/Th2 disturbance pathology.

### Unpretreated MSC did not show clear improvement of hepatic fibrosis, and TLR4+IFN-γ-activated MSC alleviated liver pathology, without increase of mortality rate; however, TLR2+IFN-γ-activated MSC displayed aggravated immunopathology

**Fig. 1:**
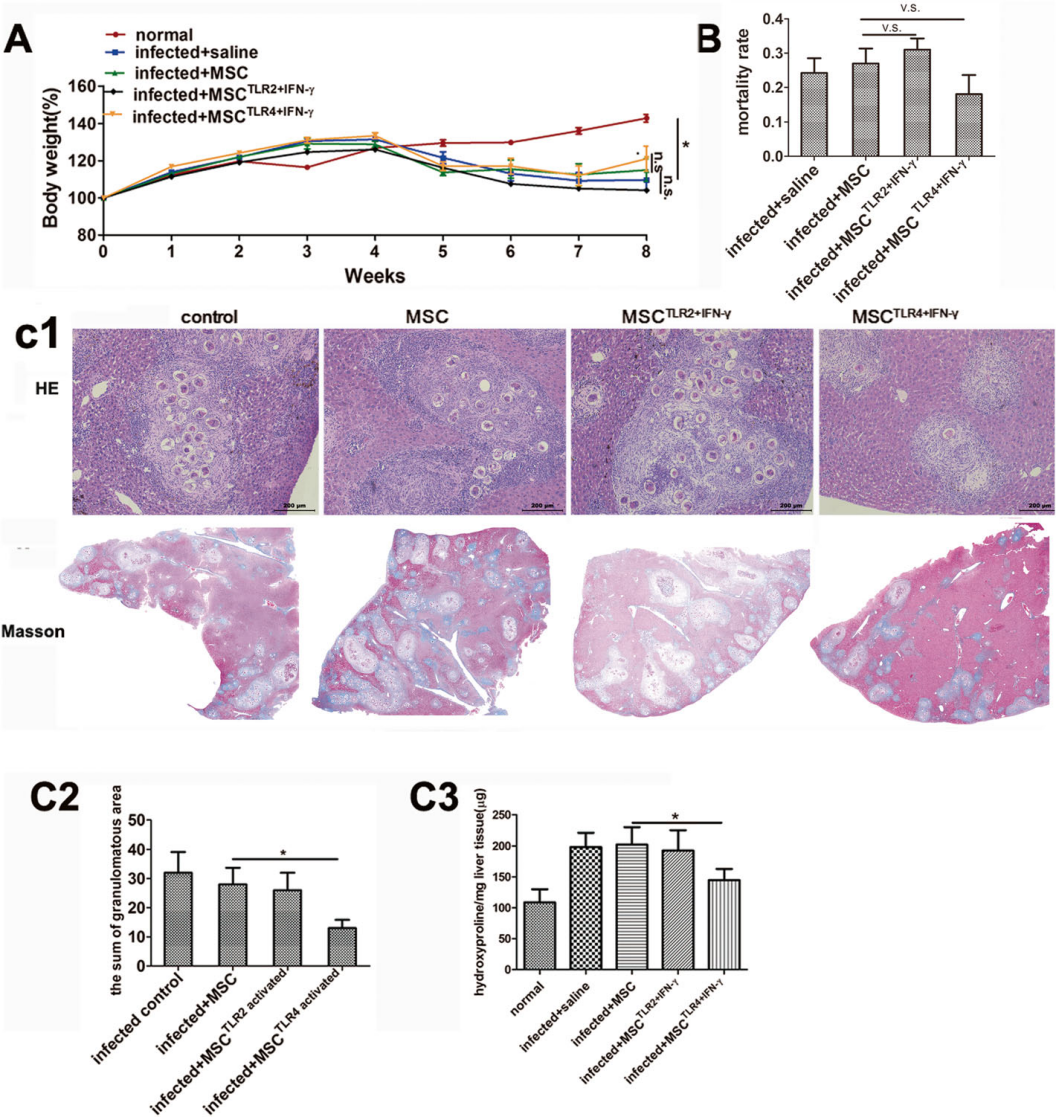
TLR4- and IFN-γ-activated MSC alleviated liver granulomatous and fibrosis in mice infected with *Schistosoma japonicum*, compared with unpretreated MSC; however, TLR2-activated MSC display aggravated immunopathology. Mice (*n* = 10 per infected group) were infected with 16 ± 2 cercariae and sacrificed 8 weeks post-infection. Body weight per week (**A**) and mortality rate (data from four repeated experiments) (**B**) were analyzed, and histological change were evaluated. Representative granulomatous was shown by HE staining and by Masson staining (**C1**), and the sum of the granulomatous area was measured by computer-assisted morphometric analysis (**C2**). Fibrosis was quantitatively compared by evaluated microgram of liver hydroxyproline per gram of the liver tissue among different groups (**C3**). Data are presented as the means ± SEM from 5 to 7 mice in each group. *P < 0.05. n.s., not significant

To examine the therapeutic potential of MSC or TLR2,4-activated MSC in vivo, we established a *Schistosoma* infection model. They were injected intravenously (i.v.) with pretreated MSC at 1 week and 3 weeks post-infection. The results showed that the body weight of infected groups clearly decreased at 5 weeks post-infection. Although the body weight did not show statistical difference among different treated MSC groups at 8 weeks, the body weight of the TLR4-activated MSC group inclined to increase a little faster (Fig. 1A). TLR4-activated MSC also alleviated liver and fibrosis after *S. japonicum* infection, without an increase in mortality rate (Fig. 1B, C).

In detail, TLR4-activated MSC group showed the lowest sum of granulomatous area (13 ± 2.86) compared with other infected groups (infected control 32 ± 7.12, MSC 28 ± 5.73, and TLR2-activated MSC 26 ± 9.02) (Fig. 1C1, C2), without an increase in mortality rate compared with the MSC group (Fig. 1B). The similar trend was observed in terms of fibrosis development, and the TLR4-activated MSC group demonstrated the mildest fibrosis shown by Masson staining and the lowest quantity of hydroxyproline per gram of the liver tissue compared with other infected groups (infected control 198.36 ± 22.63, MSC 202.32 ± 27.68, TLR2-activated MSC 192.78 ± 32.02, and TLR4-activated MSC 144.82 ± 18.22) at 8 weeks post-infection (Fig. 1C1, C3). This result indicated less deposition of collagen fibers in the liver of the TLR4-activated MSC group, suggesting that TLR4-activated MSC may be involved in the regulation of the granulomatous response to *S. japonicum* infection.

### TLR4+IFN-γ-activated MSC reduced fibrosis-related Acta2 and Col1a1, Tgfb, and Tnf level expression in the liver

**Fig. 2:**
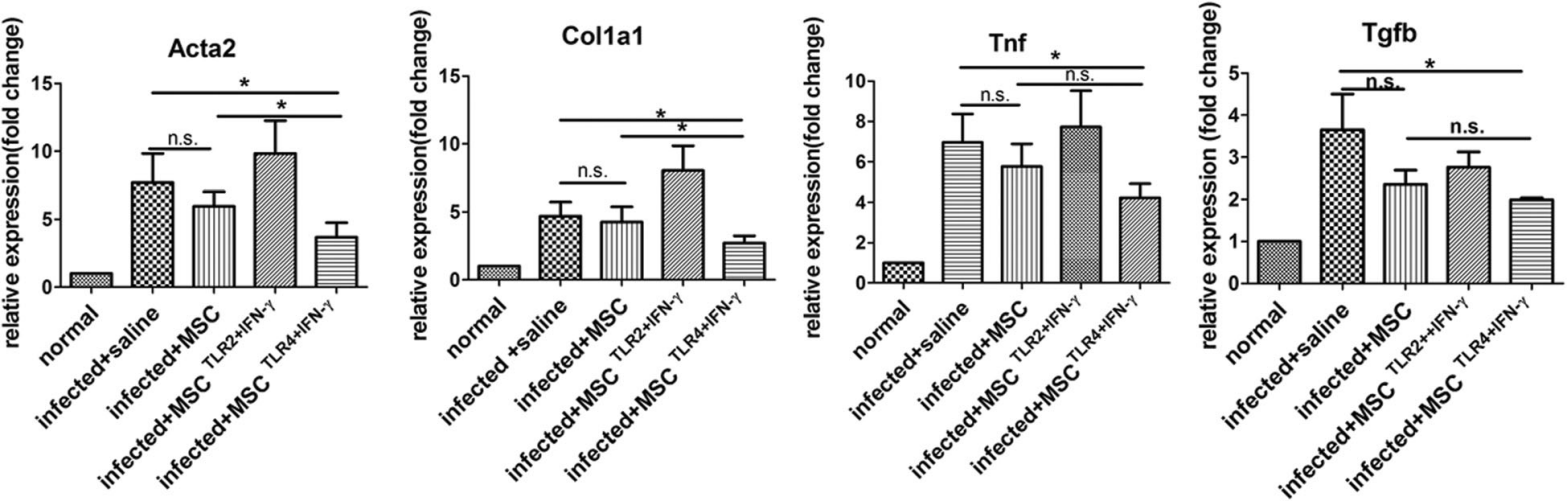
TLR4+IFN-γ-activated MSC reduced fibrosis-related Acta2, Col1a1, Tgfb, and Tnf expression in the liver. Fifty to 100 mg of liver tissue was removed from 8-week-infected mice, and the expression of Col1a1, Acta2, Tgfb, and Tnf message were assessed by semi-quantitative PCR analysis. Results were expressed as fold amplification over normal. Data are presented as the means ± SEM, *n* = 6 per group. *P < 0.05. n.s., not significant

To further ensure the effects of MSC on liver fibrosis, the gene expression of α-SMA (a symbol marker of activated hepatic stellate cell (HSC)), collagen-1 (Col-1), and fibrosis-related TGF-β in the livers of infected mice were assessed by semi-quantitative PCR. In parallel with the histology results presented above, only TLR4-activated MSC did clearly reduce Acta2, Col1a1, Tgfb, and Tnf expression in the livers compared with non-MSC control (*P* < 0.05). Moreover, compared with the MSC alone group, Acta2 and Col1a1 genes also decreased in the TLR4-activated MSC group (Fig. 2). Altogether, these results suggested treatment with TLR4-activated MSC alleviated liver fibrosis, without increasing inflammatory response.

### TLR4+IFN-γ-activated MSC increased Th1/Th2 ratio in the liver from mice infected with *Schistosoma japonicum*, and unpretreated MSC and TLR2+IFN-γ-treated MSC did not

**Fig. 3:**
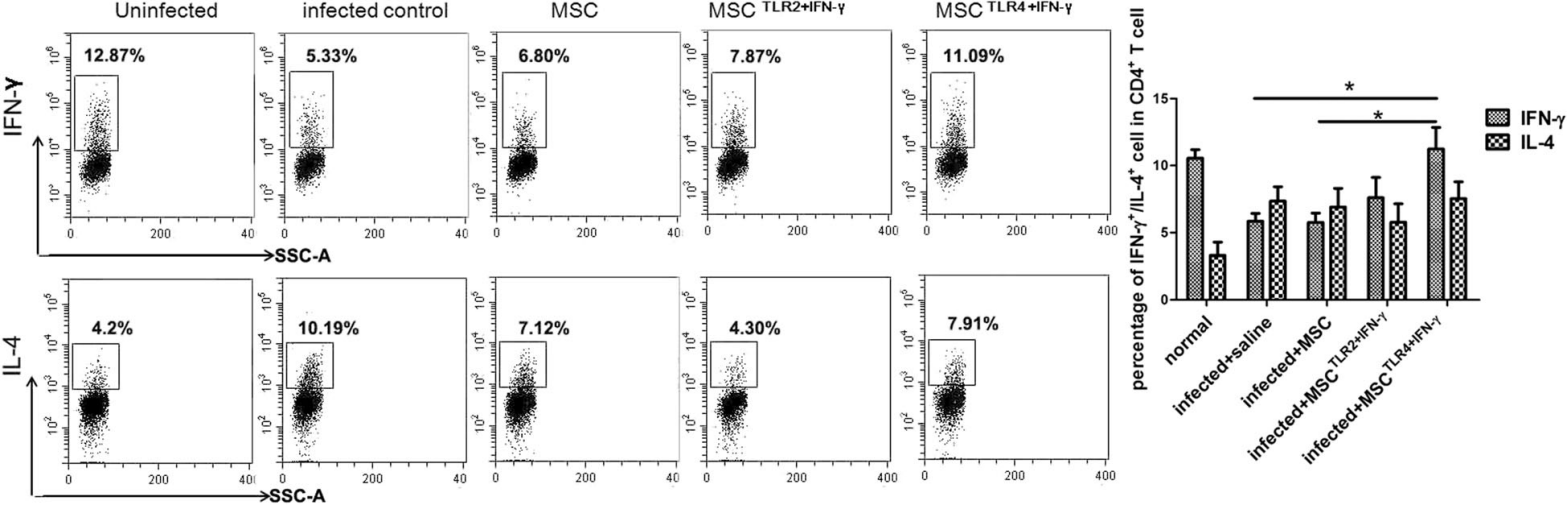
TLR4+IFN-γ-activated MSC increased CD4^+^IFN-γ^+^ T cells in the liver from mice infected with *Schistosoma japonicum*, and unpretreated MSC and TLR2+IFN-γ-treated MSC did not. The expression levels of IFN-γ and IL-4 in CD4^+^ T cells in the liver were analyzed by flow cytometry at 8 weeks post-infection (the gate strategy was shown in figure s3.). Data are presented as the means ± SEM from 7 to 10 mice in each group. *P < 0.05, compared to infected control or untreated MSC

As CD4^+^ T cells orchestrate the development of immunopathology in schistosomiasis, we investigated whether the immunopathology change resulted from the Th1/Th2 dominance. Th1 (CD3^+^CD4^+^IFN-γ^+^) and Th2 (CD3^+^CD4^+^IL-4^+^) T cells in the liver were analyzed by flow cytometry at 8 weeks post-infection. As generally reported, *Schistosoma japonicum* infection led to the inhibition of Th1 combined with an increase of Th2, compared with uninfected control and MSC alone group (*P* < 0.05) (Fig. 3). TLR4-activated MSC increased CD4^+^IFN-γ^+^ T cells in the liver. In terms of CD4^+^IL-4^+^ T cells, there is no clear difference among different groups. So, it seemed that the ratio of Th1/Th2 was recovered in the TLR4-activated MSC group.

### TLR4+IFN-γ-activated MSC group had the highest levels of IFN-γ in the serum with improved structure of the thymus and spleen, and TLR2+IFN-γ-treated MSC and unpretreated MSC showed mild increase of IFN-γ and no significant increase of IFN-γ, respectively

**Fig. 4:**
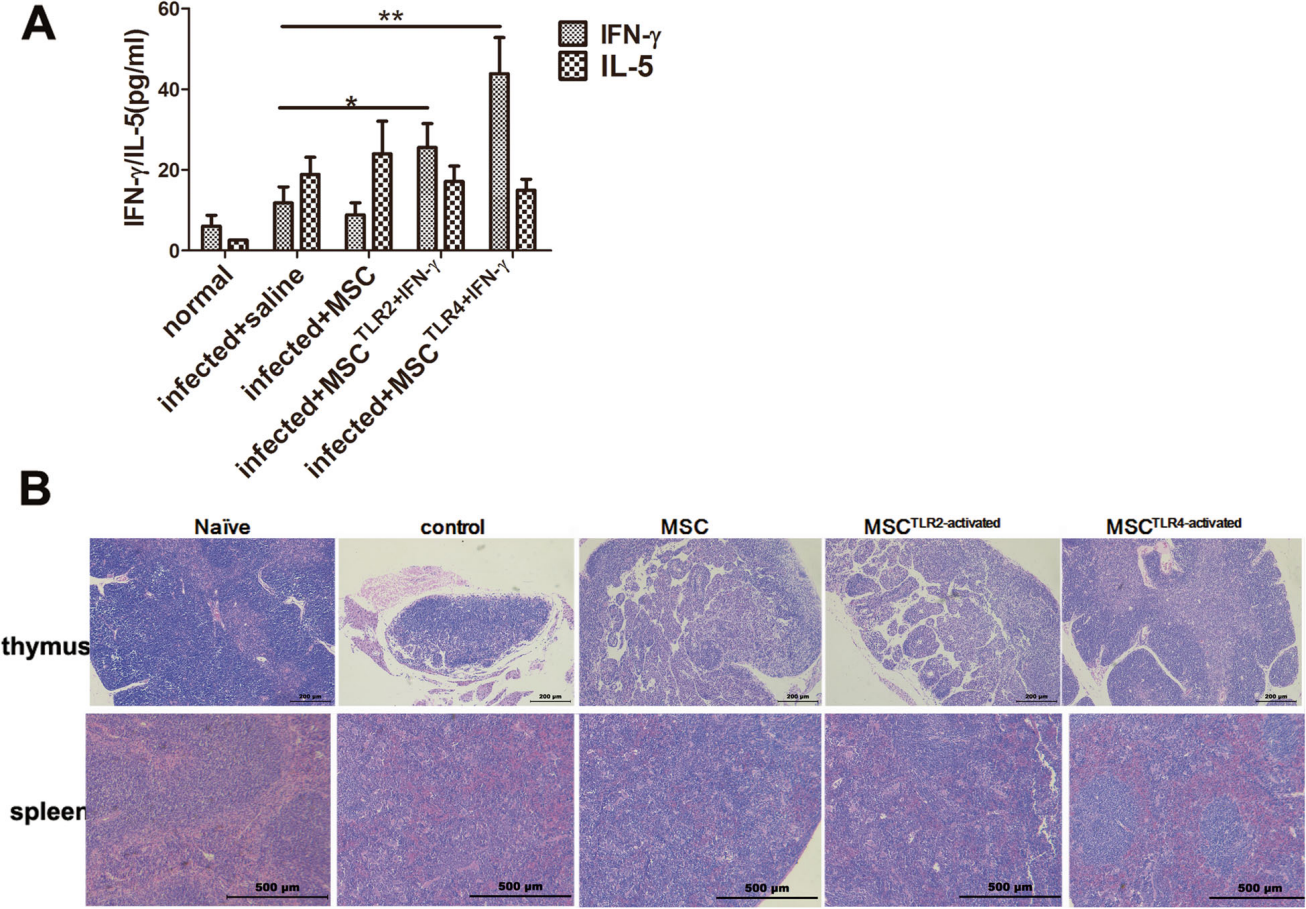
The TLR4+IFN-γ-activated MSC group had the highest levels of IFN-γ in the serum with an improved structure of the thymus and spleen, and TLR2-treated MSC and unpretreated MSC showed a mild increase of IFN-γ and no significant increase of IFN-γ, respectively. To measure systemic IFN-γ and IL-5 protein levels, the serum was taken from 8-week-infected mice and used in capture ELISAs (**a**), and histological change was evaluated by HE staining (**b**). Data are presented as the means ± SEM from 7 to 10 mice in each group. *P < 0.05; **P < 0.01

To further verify the characteristics of Th responses among different groups, we also examined the cytokine response in vivo by examining the serum levels of IFN-γ and IL-5 (by ELISA). In accord with the fluorescence-activated cell sorting (FACS) results from the liver, TLR4+IFN-γ-activated MSC increased the most systemic IFN-γ level, while maintaining comparable IL-5 level to the control. In unpretreated MSC group, a comparable level of IFN-γ and IL-5 compared to infection control was observed, and in the TLR2+IFN-γ-treated MSC group, only a medium increase of the level of IFN-γ and comparable IL-5 level to the infection control were visible (Fig. 4a).

Furthermore, in contrast to the general immuno-inhibition and structure damage to the thymus and spleen in infected mice with *Schistosoma japonicum*, LPS+ IFN-γ-activated MSC also improved the structure of the thymus and spleen. There appeared a clear corticomedullary distinction of thymus and splenic corpuscles of the spleen in LPS-activated MSC, and by contrast, cortical atrophy of the thymus and demolished splenic corpuscles in other groups (Fig. 4b). Taken together, the higher Th1 and Th2 responses in the TLR4-activated MSC group were concomitant with improved immune organ structure, while a mild Th1 dominant response in TLR2-activated MSC group and still Th2 dominant response in unpretreated MSC group did not show improvement of thymus and spleen structure.

### TLR2+IFN-γ-activated MSC most enhanced Th1 response, and correspondingly inhibited Th2 response in vitro, and TLR4+IFN-γ-activated MSC moderately increased Th1 response and decreased Th2 response

**Fig. 5:**
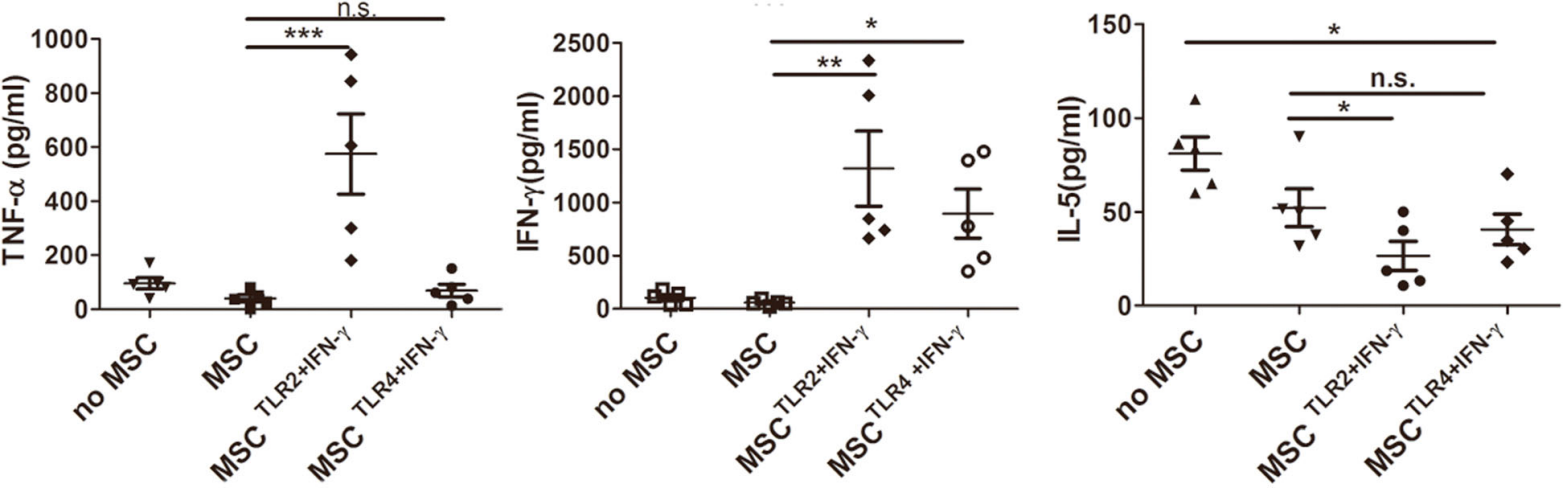
TLR2+IFN-γ-activated MSC most enhanced Th1 response, and correspondingly inhibited Th2 response in vitro, and TLR4+IFN-γ-activated MSC moderately increased Th1 response and decreased Th2 response. Spleen cells from infected mice were cultured with MSC, TLR4-activated MSC, or TLR2-activated MSC in the presence of SEA in vitro. Three days later, the culture supernatant was analyzed for IFN-γ, TNF, and IL-5 by ELISA. Results are mean ± SEM of five mice in each group and are representative of two repeat experiments. *P < 0.05; **P < 0.01; ***P < 0.001

In order to further identify the role of different kinds of MSC on the modulation of Th subsets, Th1 (IFN-γ and TNF) and Th2 cytokine(IL-5) were evaluated after spleen cells from infected mice were cultured with MSC, TLR4+IFN-γ-activated MSC, or TLR2+IFN-γ-activated MSC in vitro in the presence of SEA. The results showed that the most increase of IFN-γ and decrease of IL-5 in TLR2-activated MSC, compared with MSC; the medium increase of IFN-γ and the medium decrease of IL-5 in TLR4-activated MSC; and still low IFN-γ and the medium decrease of IL-5 in MSC group(Fig. 5). In addition, the highest level of TNF was manifest in the TLR2-activated MSC group. Thus, TLR2-activated MSC, TLR4-activated MSC, or MSC respectively induced strong, medium, or a weak Th1 response.

### TLR4+IFN-γ-activated MSC reduced the parasite egg burden, while TLR2+IFN-γ-activated MSC increased

**Fig. 6:**
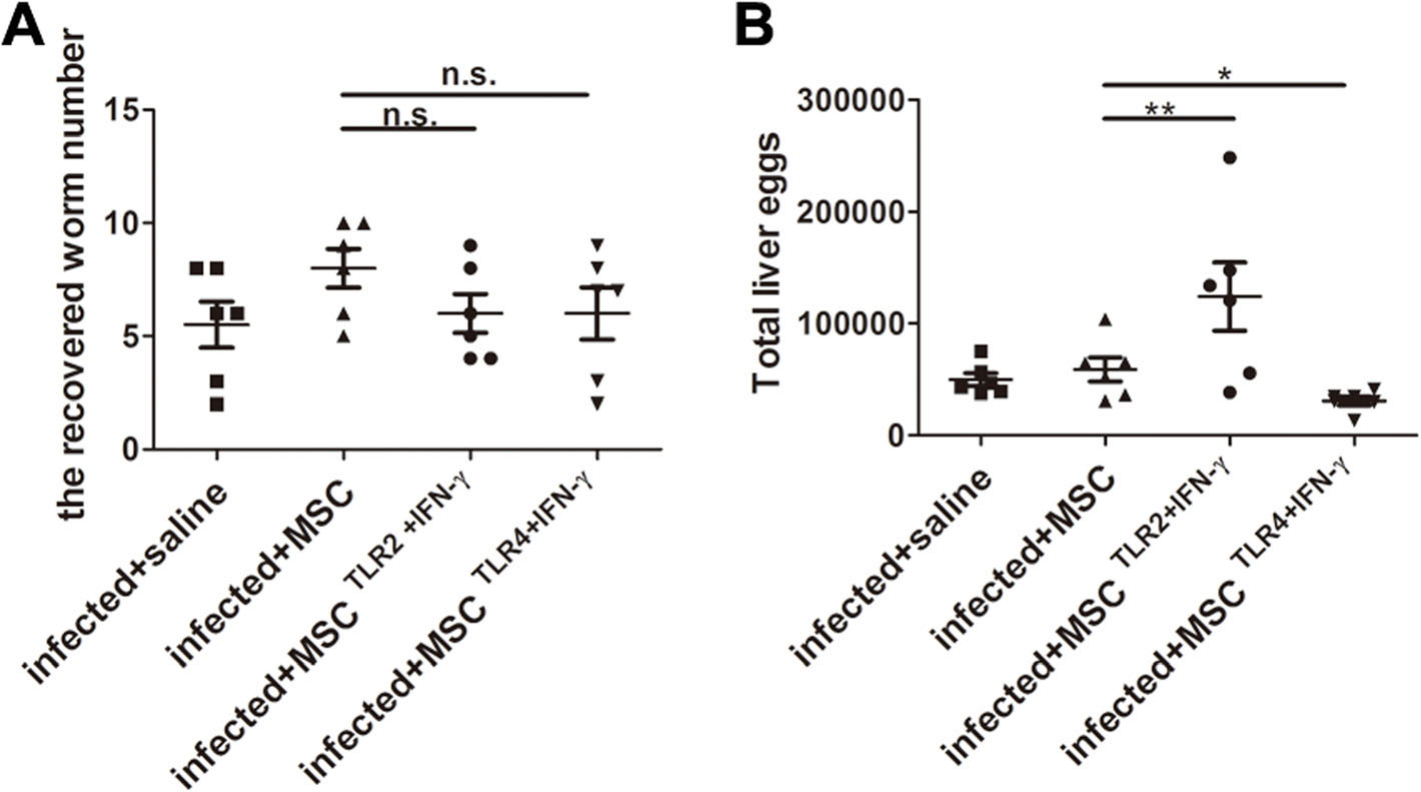
TLR4 and IFN-γ-activated MSC reduced the parasite egg burden. The average number of worms recovered from the portal vein at 8 weeks post-infection (**a**). The total number of eggs in the liver was shown in the dot plot (**b**). Each bar represents the mean ± SEM (*n* = 6). *P < 0.05; **P < 0.01. n.s., no significant

A Th1-type immune response is important for the host to clear *S. japonicum*. So, the parasite burden was also investigated. The reduced egg burden in the liver of the TLR4+IFN-γ-activated MSC group was observed, whereas the adult worm burden was comparable to the control. In contrast, increased eggs in the liver of the TLR2+IFN-γ-activated MSC group appeared (Fig. 6).

## Discussion

Recently, MSC has been used to attenuate hepatic fibrosis induced by *Schistosoma* infection which has distinct Th1/Th2 disturbance pathology [1–3]. However, the effects of MSC on the modulation of Th1/Th2 balance still waiting to be clarified. The immunoregulatory capability of MSC is largely due to the production of soluble factors which the expression varies depending on the inflammatory condition [9], and our in vitro experiment showed that TLR2 ligand or TLR4 ligand combined with IFN-gamma clearly increased key mediator (Nos2 and Il6) gene expression of MSC. In addition, Our early experiment showed that TLR2/TLR4 ligands differently effected immune modulation of MSC [20]. So, we mainly focused on the efficacy of TLR2/4 and IFN-γ-ligated MSC on the modulation of Th1/Th2 balance in the *Schistosoma* infection model.

Our results showed that TLR4**+**IFN-γ-activated MSC alleviated liver granulomatous and fibrosis in mice infected with *Schistosoma japonicum*, without a significant increase of mortality rate, and unpretreated MSC showed no clear improvement of hepatic fibrosis; however, TLR2**+**IFN-γ-activated MSC displayed aggravated immunopathology. In accord with the pathological results, TLR4**+**IFN-γ-activated MSC reduced fibrosis-related Acta2, Col1a1, and Tgfb expression in the liver. The common reason for schistosomiasis pathology attributed to Th1/Th2 dominance and IFN-γ has generally been recognized to have antifibrotic actions [2, 3, 21, 22]. Higher level of IFN-γ in the liver and serum in the TLR4**+**IFN-γ-activated MSC group indicated clear Th1 dominance in this group which partially underpinned the alleviated histological pathogenesis, and by contrast, still Th2 dominance in the unpretreated MSC group showed no clear improvement of hepatic fibrosis.

In order to further clarify the mechanism of different pretreated MSC on the pathology of schistosomiasis, their effects on Th1 (IFN-γ and TNF) and Th2 cytokine (IL-5) in vitro were evaluated by direct coculture of splenocytes from infected mice with none or different pretreated MSC in the presence of SEA. A clear Th2 dominance in the no MSC group and MSC alone group was apparent. A strong and medium Th1 responses were observed in the TLR2+IFN-γ-activated MSC group and TLR4+IFN-γ-activated MSC, respectively. This result was partially consistent with the in vivo result, in which Th2 dominance in the MSC group and Th1 dominance in the TLR2/TLR4 group were evident. However, there was still a difference between in vitro and in vivo experiments. TLR2-activated MSC did not induce the highest serum IFN-γ in vivo as it did in vitro, and TLR4 activated MSC instead. Considering the clear thymus atrophy and damage to the spleen in the TLR2-activated MSC group, we speculate that severe inflammatory response triggered by TLR2-activated MSC may significantly damage the immune organ at the acute stage of inflammation and then impair the immune response to parasite at 8 weeks post-infection, thus no improvement of liver pathology. Our finding that a high level of TNF in cultural supernatant was manifest in the TLR2-activated MSC group support this speculation. By contrast, the structure of the thymus and spleen were improved in the TLR4-activated MSC group, and even more, lowered level of TNF in vitro culture system in this group was observed (the level of TNF in vivo also showed an observed reduced trend in this group), which suggested that no extreme inflammation be triggered by TLR4-activated MSC. Therefore, although polarization to Th1 response was suggested to be involved with the increase of mortality of *Schistosoma* infection model [23], we observed alleviated liver fibrosis, without an increase of mortality in the TLR4-activated MSC group.

It was reported that atrophy of the thymic cortex was correlative to the production and dissemination of the parasite eggs in *Schistosoma*-infected mice [24]. Our results also showed an increased egg burden of the liver in the TLR2-activated MSC group or decreased egg burden in the TLR4-activated MSC group, which appeared atrophy of the thymus or improved structure of the thymus, respectively. Many results also showed the correlation of Th1-type polarization with the reduction of liver eggs [25–28]. Our results also showed that the lower egg burden appeared in the TLR4-activated MSC group which had the highest level of IFN-γ and alleviated damage to the liver. Furthermore, it was reported that IFN-γ function by activating M1 macrophage to release NO to reduce parasite burden [26, 29]. Since our results showed that TLR2/4-ligated MSC could directly induce iNOS (an enzyme that induces production of labile NO) and IL-6 expression, we wondered whether TLR4-activated MSC lowers egg burden by directly inducing NO. However, the finding that TLR2-ligated MSC producing the largest quantity of Nos2 in vitro did not lead to a decrease of egg burden in vivo, whereas TLR4-ligated MSC producing a medium level of Nos2 and highest IFN-γ in vivo led to a decrease of egg burden. This result suggested that TLR4-ligated MSC may reduce egg burden by enhancing Th1 response, not by directly inducing NO.

In conclusion, our experiment demonstrates that only the TLR4+IFN-γ-activated MSC group did show positive effects on the pathology of schistosomiasis by enhancing Th1-polarized response, which improves our knowledge about the MSC-based cell therapy for the treatment of schistosomiasis. More importantly, this work clearly indicates that different pretreated MSCs play varying roles in modulating Th1/Th2 responses, and thus showing various therapeutic effects. Therefore, when MSC is used to treat different immuno-disturbance diseases, different pretreatment methods should be seriously considered to adapt to the distinct immuno-pathogenesis of different diseases.

## Supplementary information

**Additional file 1: Figure S1.** Cell surface markers on MSC were detected by flow cytometry. **Table S1.** Primer sequences of Gapdh, Nos2, Ptgs2, Il6, Ifnb1 and Jag1 genes used in the RT-PCR. **Figure S2**. Different mRNA expression of MSCs pretreated with IFN-γ or TLR2/4 ligand alone or their combinations. **Figure S3.** A representative result from control group showed the gating strategy in Fig. 3.

## Data Availability

The data that support the findings of this study are available from the corresponding author upon reasonable request.

## Abbreviations

MSC: Mesenchymal stem cell
TLR: Toll-like receptor
α-SMA: Alpha-smooth muscle actin
Col-1: Collagen type I
TGF-β: Transforming growth factor beta
DMEM: Dulbecco’s Modified Essential Medium
PAMPs: Pathogen-associated molecular patterns
HBSS: Hanks Balanced Salt Solution
H&E: Hematoxylin and eosin
BFA: Brefeldin A
PCR: Polymerase chain reaction
IFN-γ: Interferon-γ
IL: Interleukin
HSC: Hepatic stellate cells
PGE2: Prostaglandin E2
IDO: Indoleamine 2,3-dioxygenase
NO: Nitric oxide
HGF: Hepatocyte growth factor
EAE: Experimental autoimmune encephalomyelitis
RPMI 1640: Roswell Park Memorial Institute 1640
PMA: Phorbol 12-myristate 13-acetate
ELISA: Enzyme-linked immunosorbent assay
TMB: 3,3′,5,5′-Tetramethylbenzidine
PBS: Phosphate-buffered saline
FBS: Fetal bovine serum
SEA: Soluble egg antigen
Sca-1: Stem cell antigen 1
iNOS: Inducible nitric oxide synthase
FACS: Fluorescence-activated cell sorting
