# Supplementary material for: Immunopathology in schistosomiasis is regulated by TLR2,4- and IFN-γ-activated MSC through modulating Th1/Th2 responses
Source: Stem Cell Res Ther. 2020 Jun 5;11:217. doi: 10.1186/s13287-020-01735-2 (PMC7275460; doi:10.1186/s13287-020-01735-2)
Supplement: Supplementary file 1 — Additional file 1: Figure S1. Cell surface markers on MSC were detected by flow cytometry. Table S1. Primer sequences of Gapdh, Nos2, Ptgs2, Il6, Ifnb1 and Jag1 genes used in the RT-PCR. Figure S2. Different mRNA expression of MSCs pretreated with IFN-γ or TLR2/4 ligand alone or their combinations. Figure S3. A representative result from control group showed the gating strategy in Fig. 3. [file 13287_2020_1735_MOESM1_ESM.docx]

**Supplementary material**

**
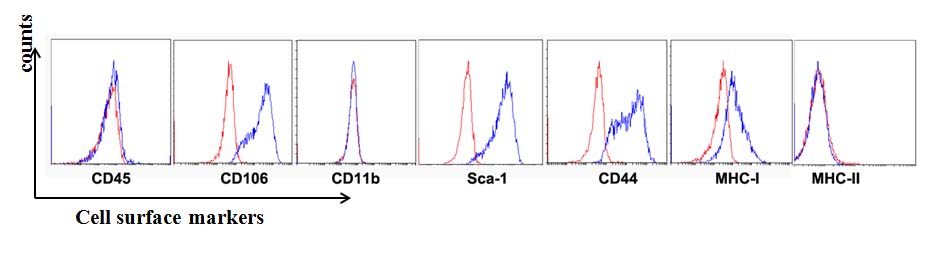
Figure s1. Cell surface markers on MSC were detected by flow cytometry.**

Table s1. Primer sequences of [Gapdh](https://www.ncbi.nlm.nih.gov/gene/14433), Nos2, [Ptgs2](https://www.ncbi.nlm.nih.gov/gene/19225), Il6, [Ifnb1](https://www.ncbi.nlm.nih.gov/gene/15977) and Jag1 genes used in the RT-PCR

|  |  | **sequence (5'→3')** |
| --- | --- | --- |
| [Gapdh](https://www.ncbi.nlm.nih.gov/gene/14433) | sense | GGCAAATTCAACGGCACAGT |
|  | Anti-sense | AGATGGTGATGGGCTTCCC |
| Nos2 | sense | TGGCCACCTTGTTCAGCTACG |
|  | Anti-sense | GCCAAGGCCAAACACAGCATA |
| [Ptgs2](https://www.ncbi.nlm.nih.gov/gene/19225) | sense | AAGCCCTCTACAGTGACATCGA |
|  | Anti-sense | GCTGATCCCGTTGATTTC |
| Il6 | sense | GCTACCAAACTGGATATAATCAGGA |
|  | Anti-sense | CCAGGTAGCTATGGTACTCCAGAA |
| Ifnb1 | sense | ACAGCCCTCTCCATCAAC |
|  | Anti-sense | CATCTTCTCCGTCATCTCC |
| Jag1 | sense | CCACGTGTTCCACAAACATC |
|  | Anti-sense | TCACAGTTCTGACCCATCCA |


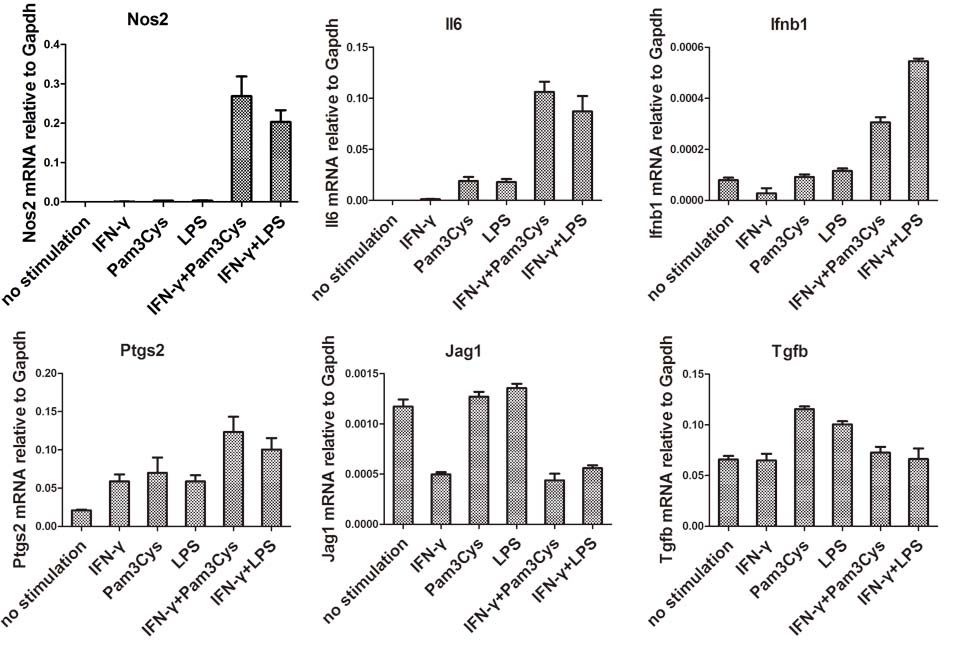
**Figure s2. Different mRNA expression of MSCs pretreated with IFN-γ or TLR2/4 ligand alone or their combinations.** MSCs were respectively incubated with IFN-γ (20ng/ml), LPS(10ng/ml), Pam3Cys(20ng/ml), or IFN-γ plus LPS, or IFN-γ plus Pam3Cys for 10 hours. The relative mRNA expression levels of Nos2, Il6, Jag1, Ptgs2, Tgfb, and Ifnb1 were analyzed by quantitative polymerase chain reaction (qPCR) and normalized with the expression of Gapdh. Data are shown as mean ± SEM (*n* = 3) from a representative of two independent experiments.

**
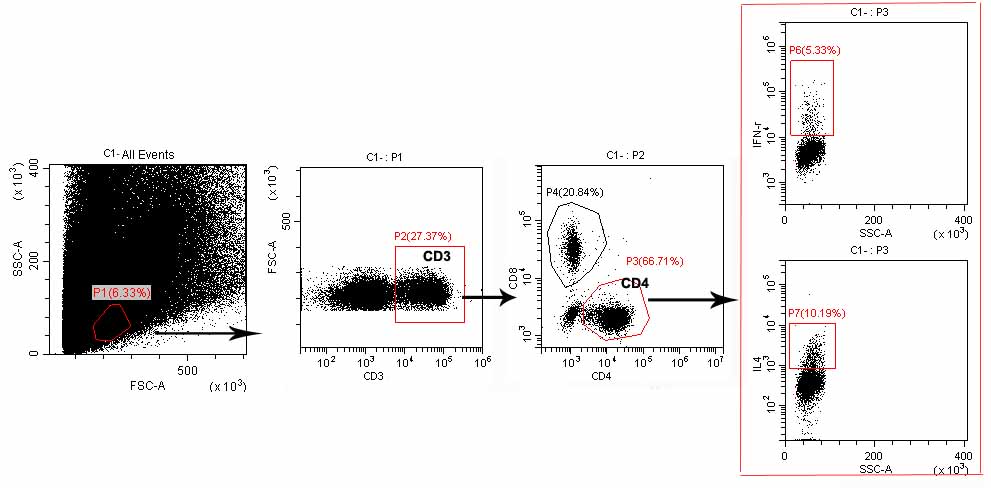
**

**Figure s3. A representative result from control group showed the gating strategy in figure3.**
